# Sleep problems in rheumatoid arthritis over 12 years from diagnosis: results from the Swedish EIRA study

**DOI:** 10.1136/rmdopen-2021-001800

**Published:** 2022-01-05

**Authors:** Lauren Lyne, Torbjörn Åkerstedt, Lars Alfredsson, Tiina Lehtonen, Saedis Saevarsdottir, Lars Klareskog, Helga Westerlind

**Affiliations:** 1 Clinical Epidemiology Division, Department of Medicine, Solna, Karolinska Institutet, Stockholm, Sweden; 2 Clinical Neuro Science, Karolinska Institute, Stockholm, Sweden; 3 Institute of Environmental Medicine (IMM), Karolinska Institute, Stockholm, Sweden; 4 Faculty of Medicine, School of Health Sciences, University of Iceland, Reykjavik, Iceland; 5 Rheumatology Division, Department of Medicine, Solna, Karolinska Institute, Stockholm, Sweden

**Keywords:** rheumatoid arthritis, epidemiology, patient reported outcome measures

## Abstract

**Objective:**

Most studies of rheumatoid arthritis (RA) and sleep have focused on established RA. We here investigate sleep quality and sleep duration in patients with newly diagnosed RA and during 1–12 years after diagnosis.

**Methods:**

Data were collected on sleep 1–12 years after diagnosis from patients diagnosed 1998–2018 in the Swedish study Epidemiological Investigation of RA. Six sleep domains (sleep problems, non-restorative sleep, insomnia, insufficient sleep, sleep quality perceived as poor and sleep considered a health problem); a global sleep score and time spent in bed were estimated. Using logistic regression, ORs were calculated for each sleep outcome by disease duration. We explored whether pain (low (Visual Analogue Scale=0–20 mm, reference), intermediate=21–70, high=71–100) or functional impairment (Health Assessment Questionnaire>1.0) was associated with problems.

**Results:**

We had sleep data on 4131 observations (n=3265 individuals). Problems with ≥1 sleep domain (global sleep score) was reported in 1578 observations (38%) and increased with disease duration (OR 1.04, 95% CI 1.02 to 1.07). Median time in bed was 8 hours (Q1-Q3: 7.5–9.0). High-grade pain increased the likelihood of sleep problems ~3–9 fold, and increased functional impairment ~4–8 fold.

**Conclusion:**

In this cohort of newly diagnosed patients with RA with access to the current treatment from diagnosis, we did not find any major problems with sleep, and existing sleep problems related mainly to pain and reduced function. Treatment of sleep problems in RA should be guided towards treating the underlying problem causing the sleep disturbance.

**Key messages**

**What is already known about this subject**

- Patients with rheumatoid arthritis (RA) have a reported high frequency of problems with sleep.
- Poor sleep has been reported to be associated with worse disease symptoms and vice versa.

**What does this study add?**

- This study follows sleep and sleep quality during the first 12 years after RA diagnosis.
- Problems with sleep were not excessive, and to a greater extent related to pain and disability than to disease duration.

**How might this impact on clinical practice or further developments?**

- Treatment of sleep problems in RA should be guided towards treating the underlying problem causing the sleep disturbance.

## Introduction

Rheumatoid arthritis (RA) is a chronic inflammatory disease in which the cardinal signs are synovitis and pain. Left untreated, the disease can progress to the point where the inflammatory process causes damage to cartilage and bone. Early diagnosis, disease-modifying antirheumatic drugs (DMARDs) and enhanced treatment strategies have improved disease outcomes, including quality of life, for patients with RA. Due to the use of such active treatment, remission is an achievable goal for many patients, the severity of long-term disability has decreased and few patients experience severe joint damage.^1^ Despite these advances patients with RA still suffer from reduced health-related quality of life and increased mortality compared with the general population.^2 3^ This may be attributed to extra-articular manifestations and comorbidities that persist despite current treatment.^4^ Constitutional symptoms such as pain, fatigue and sleep problems have also been identified as issues that might maintain a continued loss of life quality among patients with RA.^5 6^

The connection between sleep and RA in general, and pain problems in RA in particular, has been recognised for a long time. Dating back to 1970, pain during sleep and sleep disturbances was reported among 54 of 100 patients with RA when asked about sleep problems at a rheumatology clinic.^7^ In 1985, another study reported sleep disturbances in 67 out of 100 patients attending another outpatient rheumatology clinic, and almost all of these (94%) reported that the disturbances were caused by pain.^8^ Among these 100 individuals, it is worth noting that two thirds of the patients reported pain as the most important symptom of their RA. Since these studies, however, there have been important improvements in the treatment regimen in RA and more recent studies have indicated that poor control of RA contributes to poor sleep quality.^9 10^ Several studies have also examined the influence of treatment on sleep quality. An observational study from 2002 of close to 9000 patients with RA did not observe any difference in sleep quality between patients treated with antitumour necrosis factor therapies compared with those given other treatments.^10 11^ However, abatacept, another biological DMARD, was shown to have a positive effect on sleep quality in RA in two double blinded, randomised clinical trials (mean disease duration of 12 and 9 years, respectively) from the same time period.^10 11^

Studies investigating sleep problems among patients with RA have so far been performed mainly on patients with long-standing disease with only few studies investigating sleep in early RA.^12^ Moreover, large, population-based studies investigating sleep problems in patients with RA receiving standard care from diagnosis onwards, are lacking. The aim of this study was, therefore, to investigate the frequency of sleep problems in patients with RA in a cohort of Swedish patients diagnosed from 1996 and onwards, followed from diagnosis. We also aimed to investigate the trends of sleep quality during the first 12 years after RA diagnosis.

## Materials and methods

### Material

For the current study, we included patients with newly diagnosed RA according to the 1987 ACR criteria, from the Swedish population-based case–control study Epidemiological Investigation of RA (EIRA). EIRA was established in 1996 and has been extensively described elsewhere.^13 14^ In short, patients newly diagnosed with RA from clinics located in the central and southern parts of Sweden were invited to participate in EIRA. In addition to diagnosis of RA, requirements for enrollment were an age of 18 or older as well as sufficient proficiency in the Swedish language to answer a questionnaire. At diagnosis patients were asked to participate in the EIRA study and were given a questionnaire to fill out on background information and lifestyle factors. Informed consent was collected from the participants at the time of enrollment in the EIRA study. All patients received the current and active standard care at rheumatology clinics in Sweden.

In 2008 and 2009, a follow-up questionnaire with questions on lifestyle changes and patient-reported outcomes was sent to all EIRA cases who had been recruited to the EIRA study from 1996 to 2008, thus enabling cross-sectional analysis of sleep patterns at different disease durations after diagnosis of RA. After this, patients included 2009 or later have routinely been sent a follow-up questionnaire at 1 year as well as 3 years after diagnosis, thus enabling a more extensive, longitudinal, analysis of sleep patterns and development of such patterns during the first 3 years of disease. We extracted data on age, sex, year of diagnosis, disease duration (defined as the time from RA diagnosis to the time of answering the questionnaire), sleep, pain and functional impairment based on the Health Assessment Questionnaire (HAQ), from the questionnaires that were collected from October 2008 to August 2019.

A patient research partner was involved in the development of the questionnaire.

### Sleep measures and definitions of sleep problems

We defined six different sleep domains: sleep problems, non-restorative sleep, insomnia, insufficient sleep, sleep quality perceived as poor and sleep considered a health problem. The questions on sleep and the sleep domains were all based on the Karolinska Sleep Questionnaire which is an instrument for measuring subjective habitual sleep and sleepiness.^15^ The questionnaire has been validated for measuring several aspects of subjective sleep in individuals aged 18–79. The questionnaire included questions on how often the respondent had difficulties falling asleep and wakening up, had repeated awakenings with difficulties falling asleep again, did not feel well-rested at the time of awakening, woke up prematurely, had restless sleep and felt tired during the day. The questionnaire also asked whether the participants thought they were getting enough sleep, how they rated their general sleep quality, to what extent they considered sleep a health problem and what time they spent in bed.

Participants were asked to score the frequency of their sleep problems as ‘never’, ‘seldom’ (occasionally), ‘sometimes’ (several times/month), ‘often’ (1–2 times/week), ‘mostly’ (3–4 times/week) and ‘always’ (5 times/week or more). We defined sleep problems as having problems with any of falling asleep, waking up, repeated awakenings, waking up prematurely or restless sleep at least ‘mostly’; non-restorative sleep as having difficulties waking up or not feeling well-rested at the time of awakening at least ‘mostly’; insomnia as feeling tired during the day at least ‘often’ while also having problems ‘mostly’ with either difficulties falling asleep, repeated awakenings with difficulties falling asleep or waking up prematurely.

Insufficient sleep, sleep quality perceived as poor and sleep considered a health problem was defined as getting ‘clearly insufficient’ sleep (scoring ≥4 on a 5-point ordinal scale), considering their sleep as ‘quite poor’ (scoring ≥4 on a 5-point ordinal scale) and considering sleep ‘quite a big health problem’ (scoring ≥5 on a 6-point ordinal scale) respectively.

A global sleep score was calculated based on whether an individual had had a problem with at least one sleep domain. Sleep duration was estimated by calculating the average time in bed from the respondents’ reported bedtime and wake up time.

For the patients who had a sleep measure at more than one time point, (ie, that had answered both the 1-year and 3 year questionnaires), we calculated the proportion of individuals developing, never having, always having, and getting better from their sleep problems.

### Statistical analysis

In all descriptive analyses, categorical variables were described using frequencies and continuous variables using medians and the first and third quartile of the distribution (Q1, Q3). We compared categorical variables across strata by χ^2^ testing, and numerical variables by either t-test (normally distributed) or Kruskal-Wallis (non-normally distributed). Normality was assessed visually by QQ-plots.

In the main analysis, where the information from some individuals contained more than one observation, we used mixed effect models with a random intercept to account for this. We used mixed effects logistic models to assess changes in the proportion of patients experiencing sleep problems over disease duration, with disease duration as the predicting variable and the sleep domain as the outcome variable. For time in bed, we fitted a mixed effects linear regression model with disease duration as a predicting variable and time in bed as outcome variable. The logistic and linear models were adjusted for age and.

All analyses were performed in R V.3.6.

### Exploratory analysis

To further explore our results and investigate if pain, disability or short or normal long sleep duration could better explain our findings, we performed some exploratory analysis.

#### Pain and HAQ

From the EIRA questionnaire, we also extracted self-reported data on pain attributed to the joint disease during the last week, measured with the Visual Analogue Scale and HAQ. In an attempt to use clinically meaningful cutoffs rather than the distribution in our data, we categorised pain into low-grade pain (≤20 mm,^16^ reference group), intermediate pain (>20 mm to ≤70 mm), and high-grade pain (>70 mm^17^), and HAQ into low (≤1.0, reference group) and high (>1.0) disability.

To investigate if and to what extent pain or HAQ were associated with reduced sleep quality, we stratified the patients by pain and HAQ status and investigated the frequency of reported problems. Statistical significance was assessed with a logistic regression with pain or HAQ as predicting variable. We adjusted all models for age, sex and disease duration.

#### Short, normal and long time in bed

We also investigated if ‘short’ and ‘long’ sleep had an association to disease duration when compared with ‘normal’ sleep. For this, we categorised time in bed into short (<6 hours), normal (≥6 hours to ≤8 hours) and long duration (>8 hours) using cutoffs previously established in the literature,^18 19^ and investigated these per disease duration strata. We fitted a logistic regression model to assess the association between disease duration and time in bed (reference: normal time in bed). The model was adjusted for age and sex.

#### Sleep domains stratified by age and sex

We stratified each sleep domain by age (10-year intervals) and sex to investigate trends in sleep quality per age and sex strata.

### Sensitivity analysis

To detect potential differences between the questionnaires, we also performed all analyses per questionnaire (ie, the questionnaire sent in 2008/2009 with 1–12 years of follow-up, the 1-year questionnaire and the 3-year questionnaire (both started in 2010)). As this data did not include multiple observations, we used logistic regression and linear regression to estimate the ORs. We also compared the frequency of problems for each sleep domain across the questionnaires and the full cohort. Differences were assessed by calculating a 95% binomial proportion CI for the difference between two population proportions using the normal approximation method. We also compared missing data over the three cohorts and per disease duration category.

## Results

### Study population

Our final study population consisted of 4131 observations from 3265 unique individuals (2324 (71%) were female) with data on sleep from 1 to 12 years of RA duration and with a median of 3 years (Q1-Q3: 1–6), captured from November 2008 until August 2019. Participation and exclusion are in detail presented in table 1 and online supplemental figure S1.

10.1136/rmdopen-2021-001800.supp1 Supplementary data

**Table 1:** Demographics for the 3265 unique individuals newly diagnosed with RA in Sweden during 1996–2018, for the full cohort stratified per questionnaire

	Full cohort	2008/2009	1-year follow-up	3-year follow-up
N sent questionnaire	N/A	2808	1593	1499
N responded	N/A	2344	1333	1161
Participation rate (%)	N/A	83	84	77
N included in study	3265	1837	1238	1056
N women (%)	2345 (72)	1322 (72)	879 (71)	773 (73)
Median year of diagnosis (Q1–Q3)	2006 (2002–2011)	2002 (2000–2005)	2012 (2010–2015)	2011 (2009–2013)
Median age at diagnosis (Q1–Q3)	56 (46–63)	54 (44–61)	60 (48–67)	58 (47–65)
Median age at questionnaire (Q1–Q3)	61 (50–67)	61 (51–67)	61 (49–68)	61 (50–68)
Median time in bed (Q1–Q3)	8.0 (7.5–9.0)	8.0 (7.5–9.0)	8.0 (7.3–9.0)	8.0 (7.3–9.0)
	Reported problems (%)			
Total observations	4131	1837	1238	1056
Global sleep score	1578 (38)	759 (41)	444 (36)	375 (36)
Sleep problems	1010 (25)	502 (28)	274 (22)	234 (22)
Non-restorative sleep	718 (18)	349 (19)	216 (18)	153 (15)
Insomnia	454 (11)	219 (12)	117 (9)	118 (11)
Insufficient sleep	398 (10)	184 (10)	102 (8)	112 (11)
Sleep quality perceived poor	775 (19)	355 (19)	214 (17)	206 (20)
Sleep considered health problem	809 (20)	382 (21)	232 (19)	195 (19)

RA, rheumatoid arthritis.

### Sleep quality among all participants

We found that in the full cohort 1578 (38%) of the patients reported problems with at least one sleep domain. For the separate sleep domains, the participants reporting problems were 1010 (25%) for sleep problems, 718 (18%) for non-restorative sleep, 454 (11%) for insomnia, 398 (10%) for insufficient sleep, 775 (19%) for sleep quality perceived as poor and 809 (20%) for sleep considered a health problem. The median time in bed was 8.0 hours (Q1-Q3: 7.5–9.0).

### Association between disease duration and sleep quality

**Figure 1:**
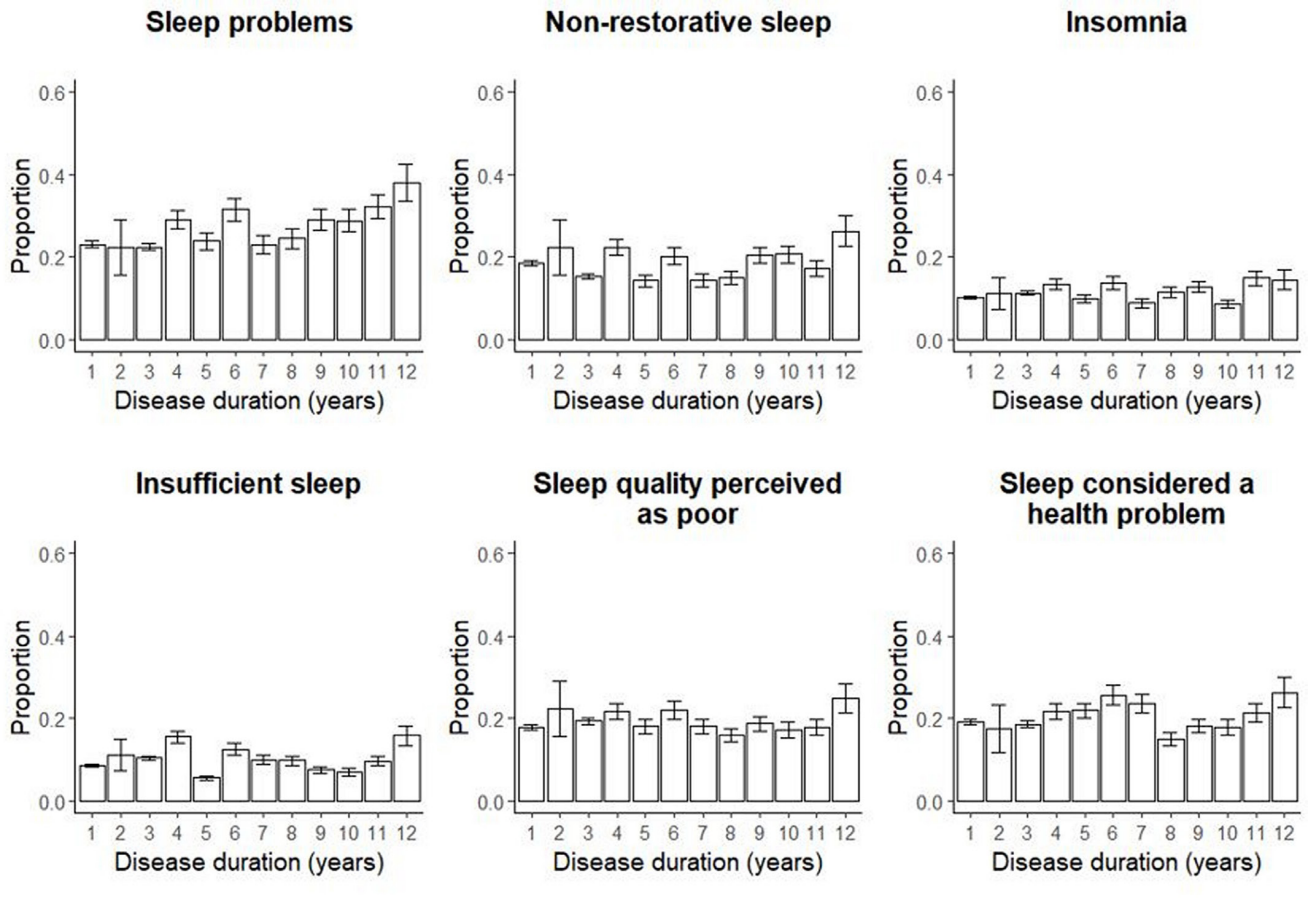
Sleep problems by domain and disease duration. Proportion of patients experiencing problems with each sleep domain stratified by disease duration using 4131 observations on sleep from 3265 individuals newly diagnosed with RA during 1996–2018 in Sweden and with a disease duration of 1–12 years at the time of data collection. Error bars represent the 95% binomial CIs calculated with the normal approximative method. RA, rheumatoid arthritis.

**Figure 2:**
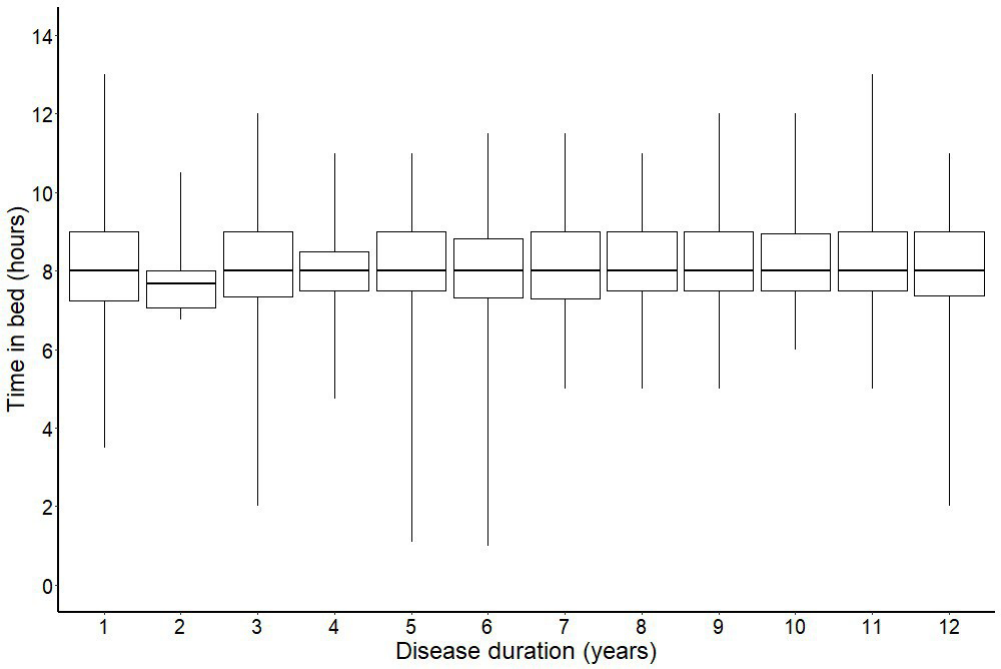
Sleep duration by disease duration. Sleep duration by disease duration using 4131 observations from 3265 individuals newly diagnosed with RA during 1996–2018 in Sweden. The boxplot represents the median, upper quartile, lower quartile, maximum and minimum values of sleep duration by disease duration. RA, rheumatoid arthritis.

Our global sleep measure (ie, experiencing problems with at least one sleep domain), increased slightly with disease duration, adjusted (aOR 1.04, 95% CI 1.02 to 1.07) (online supplemental table S1). None of the separate sleep scores (figure 1)or time in bed (figure 2) had a significant association with disease duration.

### Changes in sleep problems within individuals

For the individuals who had sleep data at both 1 and 3 years (n=866), more than half (52%, n=450) never reported any problems with sleep. The proportion reporting problems at least once varied between 15% (insufficient sleep) and 31% (sleep problems). Persistent problems ranged from 5% (insufficient sleep) and 14% (sleep problems). The full results are presented in table 2.

**Table 2:** Proportion developing/improving/never/always having problems, per sleep domain among the 866 individuals with RA newly diagnosed during 2008–2018 and that had answered the questionnaire at both 1 and 3 years after diagnosis

Sleep problems	
Always	125 (14%)
Never	597 (70%)
Improving	90 (11%)
Developing	87 (10%)
Non-restorative sleep	
Always	85 (10%)
Never	668 (78%)
Improving	89 (10%)
Developing	60 (7%)
Insomnia	
Always	58 (7%)
Never	728 (85%)
Improving	50 (7%)
Developing	57 (7%)
Insufficient sleep	
Always	41 (5%)
Never	740 (86%)
Improving	40 (5%)
Developing	51 (6%)
Sleep quality perceived as poor	
Always	100 (12%)
Never	642 (74%)
Improving	61 (7%)
Develop	66 (8%)
Sleep considered a health problem	
Always	94 (11%)
Never	635 (73%)
Improving	71 (8%)
Developing	72 (8%)

### Exploratory analysis

**Figure 3:**
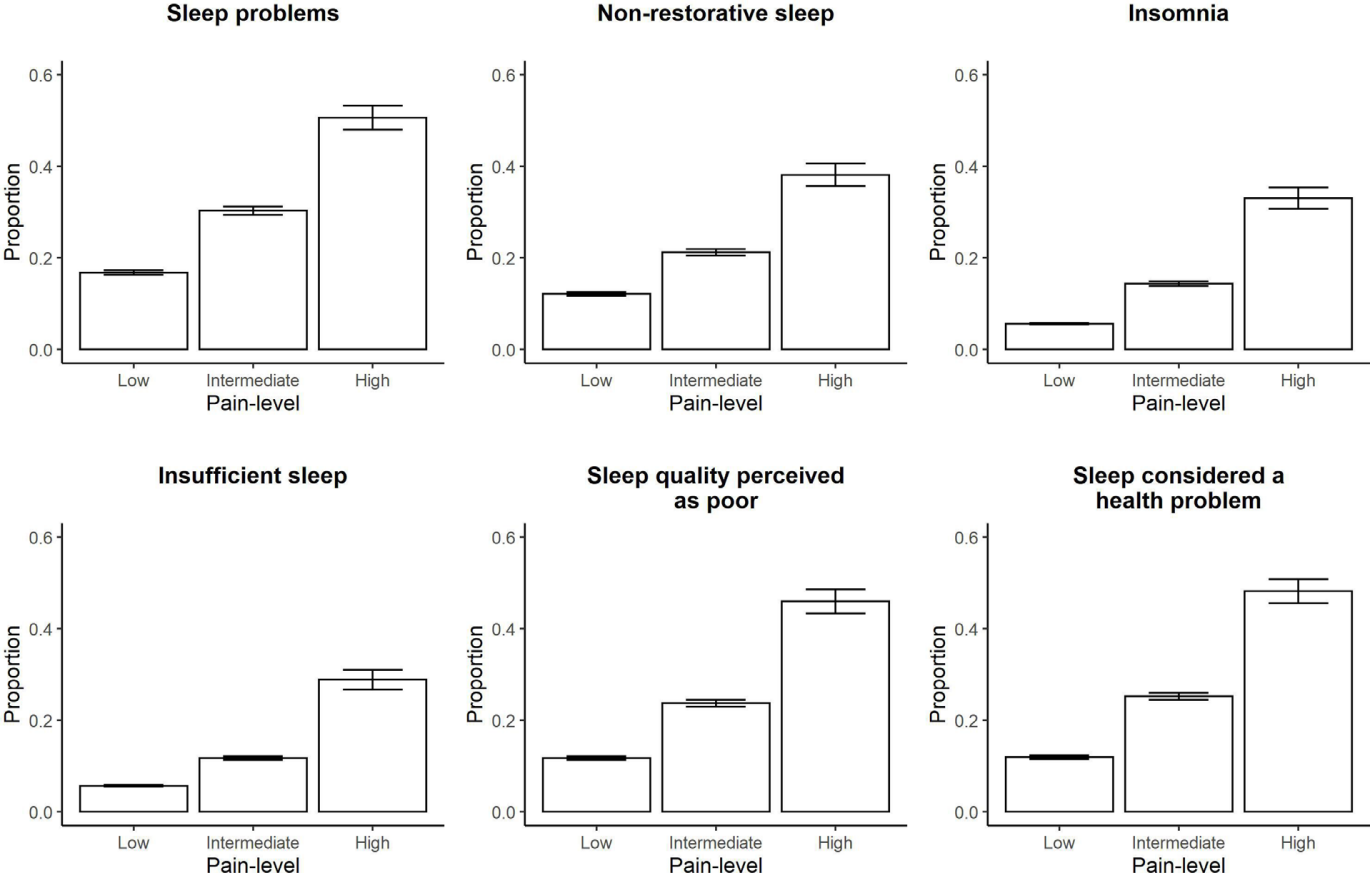
Sleep problems by pain level. Proportion of patients experiencing problems with each sleep domain stratified by pain level using 4131 observations for 3265 individuals newly diagnosed with RA during 1996–2018 in Sweden and with a disease duration of 1–12 years at the time of data collection. Error bars represent the 95% binomial CIs calculated with the normal approximative method. RA, rheumatoid arthritis.

**Figure 4:**
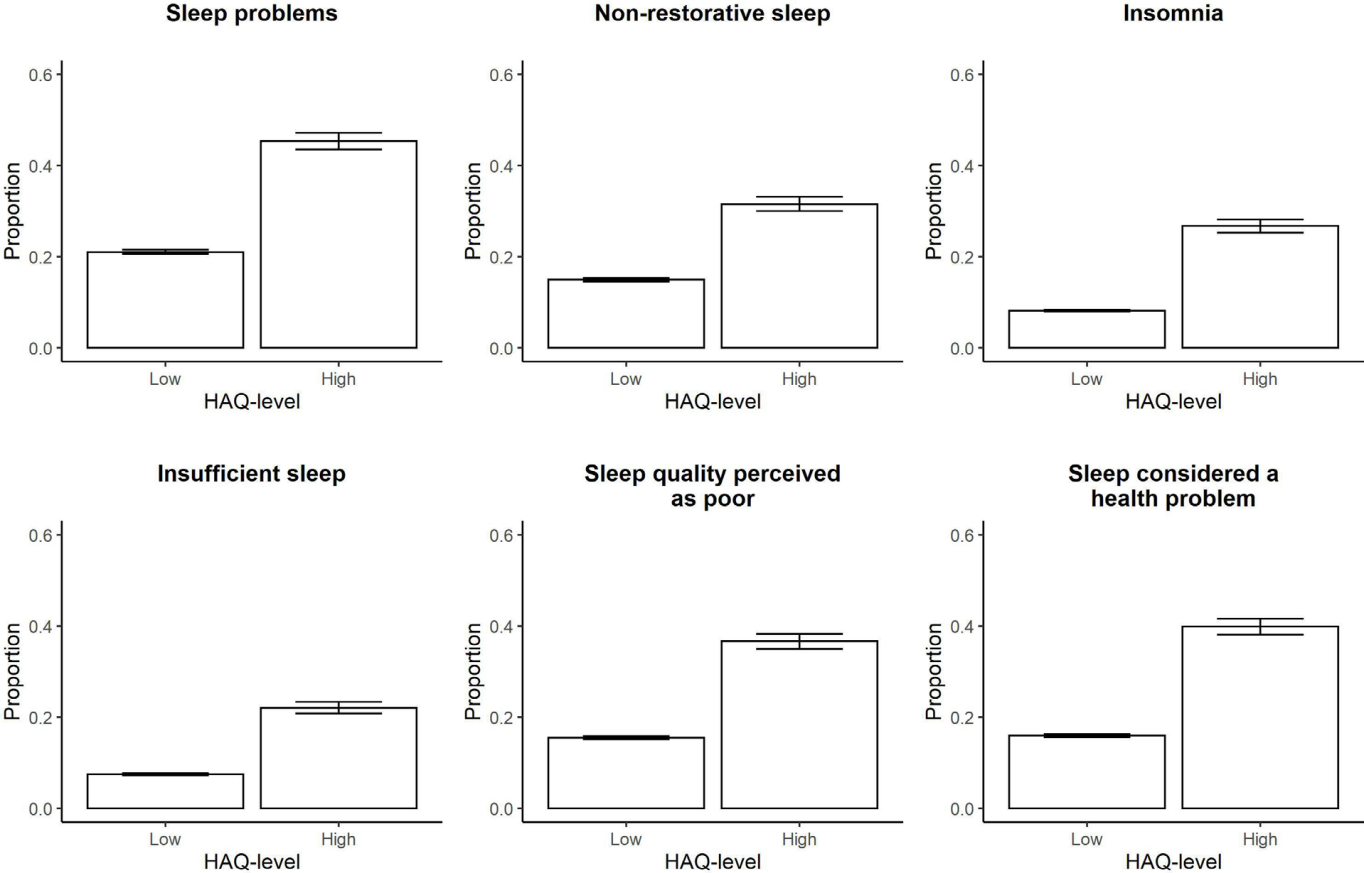
Sleep problems by disability. Proportion of patients experiencing problems with each sleep domain stratified by HAQ-level using 4131 observations for 3265 individuals newly diagnosed with RA during 1996–2018 in Sweden and with a disease duration of 1–12 years at the time of data collection. Error bars represent the 95% binomial CIs calculated with the normal approximative method. HAQ, Health Assessment Questionnaire; RA, rheumatoid arthritis.

We saw that the frequency of problems increased with pain intensity for all sleep domains (figure 3). The risk of having sleep problems was between 2 and 3 times higher in the group with intermediate pain and 3–9 times higher in the group with high-grade pain as compared with low-grade pain. Adjusting for disease duration did not weaken these associations (crude and adjusted ORs are presented in online supplemental table S2). The risk of having problems with sleep was about 4-8 times higher in the group with high HAQ-level as compared with low HAQ-level, and again disease duration did not impact these estimates (see figure 4 for proportions and online supplemental table S3 for crude and adjusted ORs).

As the proportion of individuals that had a short time in bed (<6 hours) was very small (ranging between 0% and 4%) we excluded it from further analysis (online supplemental figure S2). We did not find an association between having a long time in bed (as compared with normal time in bed) and disease duration (data not shown).

Generally, we found that problems with sleep were most frequent in the younger age-groups across all sleep domains. Problems with sleep were least frequent among the age groups 70–79 and 80–89 in most sleep domains (online supplemental figure S3).

Women reported a higher frequency of problems than men for all domains (p<0.0001) (online supplemental figure S4).

### Sensitivity analysis

Compared with the full cohort, the data from the 1-year questionnaire had a lower frequency of sleep problems (25% vs 22%, p=0.037), while the data from the 3-year questionnaire had a lower frequency of non-restorative sleep (18% vs 15%, p=0.005). The data from the 2008–2009 questionnaire had a significantly higher frequency of sleep problems (25% vs 28%, p=0.004) compared with the full cohort (table 1).

The proportion of individuals experiencing problems with at least one sleep domain as well as in the individual sleep domains did not change with disease duration for the 2008–2009 questionnaire online supplemental table S4). All ORs were close to 1. The odds of having problems increased with pain level as well as HAQ-level for all sleep domains in all questionnaires (online supplemental figures S5 and S6).

## Discussion

In this comprehensive investigation of sleep quality with 4131 observations from 3265 patients with newly diagnosed RA that were followed for up to 12 years, 39% of the patients reported some form of sleep disturbance (problems with at least one sleep domain). Women reported a higher frequency of sleep problems than men and the oldest age groups (70–89 years old) in general reported the lowest proportion of sleep problems. Furthermore, the frequency of problems based on the global sleep score increased with disease duration, while median time in bed did not change. However, pain and functional impairment had the strongest associations to problems with sleep and to a large extent explained our findings.

Early studies of sleep in RA reported higher frequencies of problems with sleep than we found in this study.^7 8^ These studies included patients with established RA in the 1970s and 1980s, recruited at rheumatology clinics. Since these studies, the treatment of RA has changed dramatically, and disease remission and pain remission are now achievable goals in many patients. Further, today’s standard of care with early diagnosis, treat to target approach, and aiming to maintain disease remission, may prevent patients from ever developing severe pain conditions and disability, the two factors with the strongest association to sleep problems in our present study. In this project, we investigated sleep in a cohort of Swedish patients during the years 2008–2019, that is, they were all able to be treated with synthetic (mainly methotrexate) and biological DMARDs. As the healthcare system in Sweden is tax funded, supporting equal access to care and treatment, individuals in the cohort investigated here has both had the opportunity to be treated with modern anti-rheumatic drugs and to receive treatment according to needs from diagnosis and onwards. An explanation to the relatively limited sleep problems in this group of patients as compared with patients in several previously published studies may, therefore, be better control of the RA disease.

Further, our cohort involves patients newly diagnosed with RA, followed from diagnosis and up to twelve years, with a response rate of 82%. Thus, our lower observed frequency of problems with sleep compared with previous studies, might also in part be attributed to the minimal inclusion bias in our cohort due to the very low loss of participants.

In this project, we extend on previous reports by investigating sleep problems over time, both within the same individual and cross-sectional in our cohort. We observed that disease duration had a very weak association with sleep problems, and that there was little variation within individuals in their reporting of sleep problems. Further, we did not observe a higher frequency of sleep problems among individuals with longer disease duration at the time point of the questionnaire, compared with those answering 1 and 3 years after diagnosis. In total, this speaks for little fluctuation in sleep problems over time and supports the observation that disease duration does not have a strong association with sleep problems.

Our global sleep score was not the only measure deviating from previously published data. Insomnia has been studied in the context of RA, with frequencies as high as 63%.^20 21^ Although the mean disease duration was longer in the study by Mustafa *et al*^20^ compared with our present study (7.8 years vs 4.0 years), this is not enough to explain the differences, as we in our study did not observe that the rate of insomnia changed with disease duration. Worth noting here is that the insomnia measure used in our present study is clinically validated, and that our observed 11% of insomnia, is, in fact, more in line with the frequency of insomnia in the general Swedish population.^22^ However, other possible explanations to the difference might be attributed to differences in the frequency of insomnia between country populations, as well as possible differences in the severity of disease between study populations.

Strengths of the present study include the use of an established questionnaire for measuring sleep quality that has been validated against normative data and is based on clinically standardised measures such as the diagnostic and statistical manual of mental disorders’ criteria for insomnia.^15 23^ To the best of our knowledge, this is the largest study investigating sleep problems in early RA and the first studying how sleep quality changes with time since onset of disease. We follow a well-characterised cohort of patients with RA from diagnosis, all receiving modern standard care at rheumatology clinics in Sweden, and with low loss to follow-up. The findings should be generalisable to the Swedish population and populations receiving similar care, that is, most RA populations in high-income countries nowadays.

However, there are also limitations that warrant some consideration. First, the questionnaire data only allowed us to capture reported time in bed, a subjective sleep measure. Although a limitation of the study is that we did not also have an objective measure of sleep, such as polysomnography, it should be noted that a subjective measure of sleep is still of importance. Even if self-reported time in bed cannot fully replace an objective measure, it can be seen as an indicator of poor sleep quality and permits measurement in larger population sizes.^24^ Second, we did not adjust for comorbid conditions such as depression that have a known correlation with insomnia.^25 26^

In conclusion, we present here a large study on sleep in early RA and how sleep quality changes with disease duration. We find that problems with sleep in this modern-day cohort with access to effective treatment from diagnosis is not excessive and have decreased since early reports. We also find that having some form of problem with sleep and sleep problems increased with disease duration, but that reduced sleep quality to a much greater extent seemed to relate to disease measures such as pain and functional impairment. Treatment of sleep problems in RA should, therefore, be guided towards treating the underlying problem, such as pain.

## Data Availability

Due to ethical permission, data from EIRA cannot be publicly shared. Please contact the principal investigators for data requests for applicable studies. For further information go to http://www.eirasweden.se/Kontakt_EIRA.htm.
