# Supplementary material for: Sleep problems in rheumatoid arthritis over 12 years from diagnosis: results from the Swedish EIRA study
Source: RMD Open. 2022 Jan 5;8(1):e001800. doi: 10.1136/rmdopen-2021-001800 (PMC8734013; doi:10.1136/rmdopen-2021-001800)

Supplementary

This document contains supplementary information for Lyne et al “Sleep in early rheumatoid arthritis”

Contents

Supplementary results

Supplementary Tables S1-S8

Supplementary Figures S1-S6

Supplementary Table S1: Crude and adjusted ORs with disease duration as exposure and sleep domain as outcome in the full cohort with 4131 observations of sleep data from 3265 patients with early RA diagnosed during 1996-2018 in Sweden.

	**N (% reporting problems)**	**Crude odds ratio**	**Adjusted odds ratio***
**At least one type of problem (global sleep measure)**	*1578 (38)*	1.04 (1.01-1.06)	1.04 (1.02-1.07)
**Sleep problems**	*1010 (25)*	1.08 (1.00-1.18)	1.09 (1.00-1.18)
**Non-restorative sleep**	*718 (18)*	0.98 (0.88-1.09)	0.99 (0.89-1.10)
**Insomnia**	*454 (11)*	1.09 (0.97-1.23)	1.10 (0.98-1.24)
**Insufficient sleep**	*398 (10)*	1.10 (0.97-1.24)	1.10 (0.98-1.25)
**Sleep quality perceived as poor**	*775 (19)*	1.06 (0.96-1.16)**	1.06 (0.97-1.17)
**Sleep considered a health problem**	*809 (20)*	1.05 (0.96-1.15)	1.07 (0.97-1.17)

*Adjusted for sex and age at the time of answering the follow-up questionnaire
** Adjusted for sex as the fully crude model did not converge.

Supplementary Table S2: The crude and adjusted ORs with pain-level as exposure and sleep domain as outcome in full cohort of 3265 individuals diagnosed with RA during 1996-2018 in Sweden and with a disease duration of 1-12 years at the time of data collection.

	Pain-level	N (% reporting problems)	Crude odds ratio	Adjusted odds ratio*
**Sleep problems**	Low	349 (17)	Ref	Ref
	Intermediate	486 (30)	1.63 (0.96-2.76)	1.57 (0.93-2.67)
	High-grade	124 (51)	3.42 (1.27-9.26)	3.23 (1.19-8.78)
**Non-restorative sleep**	Low	252 (12)	Ref	Ref
	Intermediate	342 (21)	1.93 (1.02-3.65)	1.91 (1.01-3.62)
	High-grade	93 (38)	8.40 (3.18-22.22)	8.44 (3.13-22.78)
**Insomnia**	Low	117 (6)	Ref	Ref
	Intermediate	232 (14)	2.92 (1.30-6.52)	2.85 (0.93-8.70)
	High-grade	81 (33)	3.39 (0.85-13.50)	3.22 (0.63-16.39)
**Insufficient sleep**	Low	119 (6)	Ref	Ref
	Intermediate	191 (12)	3.09 (1.21-7.88)	0.83 (0.28-2.47)
	High-grade	71 (29)	18.65 (5.82-59.71)	7.63 (1.45-40.04)
**Sleep quality perceived as poor**	Low	246 (12)	Ref	Ref
	Intermediate	385 (24)	2.64 (1.39-5.01)	2.55 (1.34-4.85)
	High-grade	113 (46)	9.22 (3.38-25.14)	8.73 (3.18-23.94)
**Sleep considered a health problem**	Low	250 (12)	Ref	Ref
	Intermediate	409 (25)	3.01 (1.61-5.62)	2.73 (2.73-2.73)
	High-grade	118 (48)	14.90 (5.64-39.38)	8.92 (5.98-13.30)

*Adjusted for sex, age at the time of answering the questionnaire and disease duration.

Supplementary Table S3: The crude and adjusted ORs with HAQ > 1 as exposure and sleep domain as outcome in full cohort of 3265 individuals diagnosed with RA during 1996-2018 in Sweden and with a disease duration of 1-12 years at the time of data collection.

	HAQ-level	N (% reporting problems)	Crude odds ratio	Adjusted odds ratio*
**Sleep problems**	Low	712 (21)	REF	REF
	High	227 (45)	3.80 (1.81-7.99)	3.59 (1.68-7.68)
**Non-restorative sleep**	Low	507 (15)	REF	REF

	High	159 (32)	5.03 (2.38-10.62)	6.09 (2.73-13.59)
**Insomnia**	Low	279 (8)	REF	REF
	High	135 (27)	3.49 (1.34-9.11)	4.40 (2.11-9.18)
**Insufficient sleep**	Low	255 (7)	REF	REF
	High	112 (22)	6.93 (2.85-16.84)	6.86 (6.80-6.93)
**Sleep quality perceived as poor**	Low	528 (15)	REF	REF
	High	186 (37)	4.39 (2.02-9.56)	4.20 (1.89-9.37)
**Sleep considered a health problem**	Low	542 (16)	REF	REF
	High	202 (40)	7.04 (3.41-14.53)	8.18 (3.75-17.81)

*Adjusted for sex, age at the time of answering the questionnaire and disease duration.

Supplementary Table S4: The crude and adjusted ORs with disease duration as exposure and sleep domain as outcome in the subcohort consisting of 1837 individuals newly diagnosed with RA during 1996-2008 and that answered the 2008-2009 questionnaire.

	N (% reporting problems)	Crude odds ratio	Adjusted odds ratio*
**At least one type of problem**	759 (42)	1.00 (0.97-1.03)	1.01 (0.98-1.04)
**Sleep problems**	502 (28)	1.03 (0.99-1.06)	1.03 (1.00-1.07)
**Non-restorative sleep**	349 (19)	0.99 (0.95-1.02)	1.00 (0.96-1.04)
**Insomnia**	219 (12)	0.99 (0.95-1.04)	1.00 (0.96-1.05)
**Insufficient sleep**	184 (10)	0.98 (0.93-1.03)	0.99 (0.94-1.04)
**Sleep quality perceived as poor**	355 (19)	0.98 (0.95-1.02)	0.99 (0.95-1.03)
**Sleep considered a health problem**	382 (21)	0.99 (0.95-1.03)	1.01 (0.97-1.05)

* Adjusted for sex and age at the time of answering the questionnaire.

Supplementary Table S5: The crude and adjusted ORs with pain-level as exposure and sleep domain as outcome in the subcohort consisting of the 1837 individuals newly diagnosed with RA during 1996-2008 and that answered the 2008-2009 questionnaire.

	2008-2009			1-year follow-up			3-year follow-up		
Pain-level	N (%) with problems	Crude OR	Adjusted OR*	N (%) with problems	Crude OR	Adjusted OR*	N (%) with problems	Crude OR	Adjusted OR*
**Sleep problems**									
Low	140 (18)	Ref	Ref	105 (16)	Ref	Ref	104 (17)	Ref	Ref
Intermediate	258 (34)	2.4 (1.9-3.0)	2.3 (1.8-2.9)	135 (27)	2.0 (1.5-2.6)	1.9 (1.4-2.5)	93 (28)	1.9 (1.4-2.6)	1.8 (1.3-2.5)
High-grade	57 (52)	5.1 (3.3-7.7)	4.8 (3.2-7.4)	34 (52)	5.7 (3.4-9.7)	5.4 (3.2-9.3)	33 (47)	4.5 (2.7-7.5)	4.2 (2.5-7.0)
**Non-restorative sleep**									
Low	101 (13)	Ref	Ref	85 (13)	Ref	Ref	66 (11)	ref	ref
Intermediate	179 (23)	2.1 (1.6-2.7)	2.0 (1.6-2.7)	102 (20)	1.7 (1.3-2.4)	1.7 (1.2-2.3)	61 (18)	1.9 (1.3-2.7)	1.8 (1.3-2.7)
High-grade	41 (37)	4.1 (2.7-6.4)	3.9 (2.5-6.1)	28 (43)	5.1 (3.0-8.7)	5.1 (2.9-8.9)	24 (35)	4.5 (2.6-7.8)	4.3 (2.5-7.7)
**Insomnia**									
Low	47 (6)	Ref	Ref	33 (5)	Ref	Ref	37 (6)	Ref	Ref
Intermediate	111 (14)	2.7 (1.9-3.9)	2.7 (1.9-3.8)	66 (13)	2.9 (1.9-4.5)	2.7 (1.8-4.2)	55 (16)	3.0 (2.0-4.7)	3.0 (1.9-4.7)
High-grade	70 (36)	9.2 (5.7-15.0)	9.0 (5.5-14.7)	18 (28)	7.3 (3.8-13.8)	6.8 (3.5-13.2)	23 (33)	7.7 (4.3-14.1)	7.3 (4.0-13.5)
**Insufficient sleep**									
Low	50 (6)	Ref	Ref	32 (5)	ref	ref	37 (6)	ref	ref
Intermediate	91 (12)	2.0 (1.4-2.9)	1.9 (1.3-2.8)	48 (10)	2.1 (1.3-3.3)	2.0 (1.2-3.1)	52 (15)	2.9 (1.8-4.5)	2.8 (1.8-4.4)
High-grade	27 (24)	4.9 (2.9-8.2)	4.6 (2.7-7.7)	22 (34)	10.0 (5.4-18.8)	9.5 (5.0-17.9)	22 (31)	7.3 (4.0-13.4)	7.0 (3.8-12.9)
**Sleep quality perceived as poor**									
Low	92 (11)	Ref	Ref	72 (11)	Ref	Ref	82 (13)	Ref	Ref
Intermediate	188 (24)	2.5 (1.9-3.2)	2.4 (1.8 -3.1)	110 (22)	2.3 (1.7-3.2)	2.2 (1.6 -3.0)	87 (26)	2.3 (1.6-3.2)	2.2 (1.6 -3.1)
High-grade	49 (44)	6.1 (4.0-9.5)	5.8 (3.8-9.0)	32 (49)	7.9 (4.6-13.7)	7.7 (4.4-13.3)	32 (46)	5.6 (3.3-9.5)	5.3 (3.1-8.9)
**Sleep considered a health problem**									
Low	99 (12)	Ref	Ref	75 (11)	Ref	Ref	76 (12)	Ref	Ref
Intermediate	202 (26)	2.5 (1.9-3.3)	2.4 (1.9-3.2)	125 (25)	2.6 (1.9-3.5)	2.5 (1.8-3.5)	82 (24)	2.3 (1.6-3.3)	2.3 (1.6-3.3)
High-grade	53 (48)	6.5 (4.3-10.0)	6.3 (4.1-9.8)	32 (49)	7.6 (4.4-13.0)	7.7 (4.4-13.4)	33 (48)	6.7 (3.9-11.3)	6.4 (3.7-10.9)

*Adjusted for sex and age at the time of answering the questionnaire. The 2008/2009 subcohort was additionally adjusted for disease duration.

Abbreviations: OR, odds ratio.

Supplementary Table S6: The crude and adjusted ORs with HAQ-level as exposure and sleep domain as outcome in the tree cohorts consisting of those that answered the questionnaire in 2008-2009, the questionnaire at 1 year after diagnosis, and at 3 years after diagnosis.

	**2008–2009**			**1-year follow-up**			**3-year follow-up**		
	N (%) with problems	Crude OR	Adjusted OR*	N (%) with problems	Crude OR	Adjusted OR*	N (%) with problems	Crude OR	Adjusted OR*
**Sleep problems**									
Low	329 (23)	Ref	Ref	209 (20)	Ref	Ref	174 (19)	Ref	Ref
High	125 (46)	2.8 (2.1-3.7)	2.7 (2.0-3.5)	55 (44)	3.3 (2.2-4.8)	3.3 (2.2-4.9)	47 (45)	3.4 (2.3-5.2)	3.3 (2.2-5.1)
**Non-restorative sleep**									
Low	232 (16)	Ref	Ref	163 (15)	Ref	Ref	112 (12)	Ref	Ref
High	82 (30)	2.2 (1.6-2.9)	2.2 (1.7-3.0)	44 (35)	3.1 (2.0-4.5)	3.4 (2.2-5.2)	33 (32)	3.3 (2.1-5.2)	3.6 (2.2-5.8)
**Insomnia**									
Low	123 (9)	Ref	Ref	80 (7)	Ref	Ref	76 (8)	Ref	Ref
High	69 (25)	3.5 (2.5-4.9)	3.8 (2.7-5.3)	33 (26)	4.4 (2.8-7.0)	4.7 (2.9-7.6)	33 (32)	5.0 (3.1-8.1)	5.2 (3.2-8.6)
**Insufficient sleep**									
Low	109 (8)	Ref	Ref	67 (6)	Ref	Ref	79 (9)	Ref	Ref
High	57 (20)	3.1 (2.2-4.4)	3.2 (2.2-4.5)	29 (23)	4.5 (2.8-7.4)	4.7 (2.8-7.7)	26 (25)	3.5 (2.1-5.8)	3.6 2.1-6.0)
**Sleep perceived as poor**									
Low	218 (15)	Ref	Ref	157 (15)	Ref	Ref	153 (17)	Ref	Ref
High	100 (36)	3.2 (2.3-4.1)	3.0 (2.3-4.0)	47 (38)	3.5 (2.4-5.2)	3.7 (2.5-5.6)	39 (38)	3.0 (1.9-4.6)	2.8 (1.8-4.4)
**Sleep considered a health problem**									
Low	230 (16)	Ref	Ref	172 (16)	Ref	Ref	140 (15)	Ref	Ref
High	113 (41)	3.6 (2.7-4.7)	3.9 (2.9-5.2)	47 (38)	3.2 (2.1-4.7)	3.6 (2.4-5.4)	42 (40)	3.7 (2.4-5.7)	3.9 (2.5-6.1)

* Adjusted for sex and age at the time of answering the questionnaire. The 2008/2009 subcohort was additionally adjusted for disease duration.
Abbreviations: OR, odds ratio.

Figure S1: Flowchart of the inclusion and exclusion of patients in each analysis. The light gray boxes represent individuals that were excluded completely from analysis due to either incorrect diagnosis, retracted consent, or missing data on predicting variables as well as covariates. The dark gray boxes represent the number of missing values for each sleep domain, which were excluded in the descriptive statistics and analysis with each individual domain.

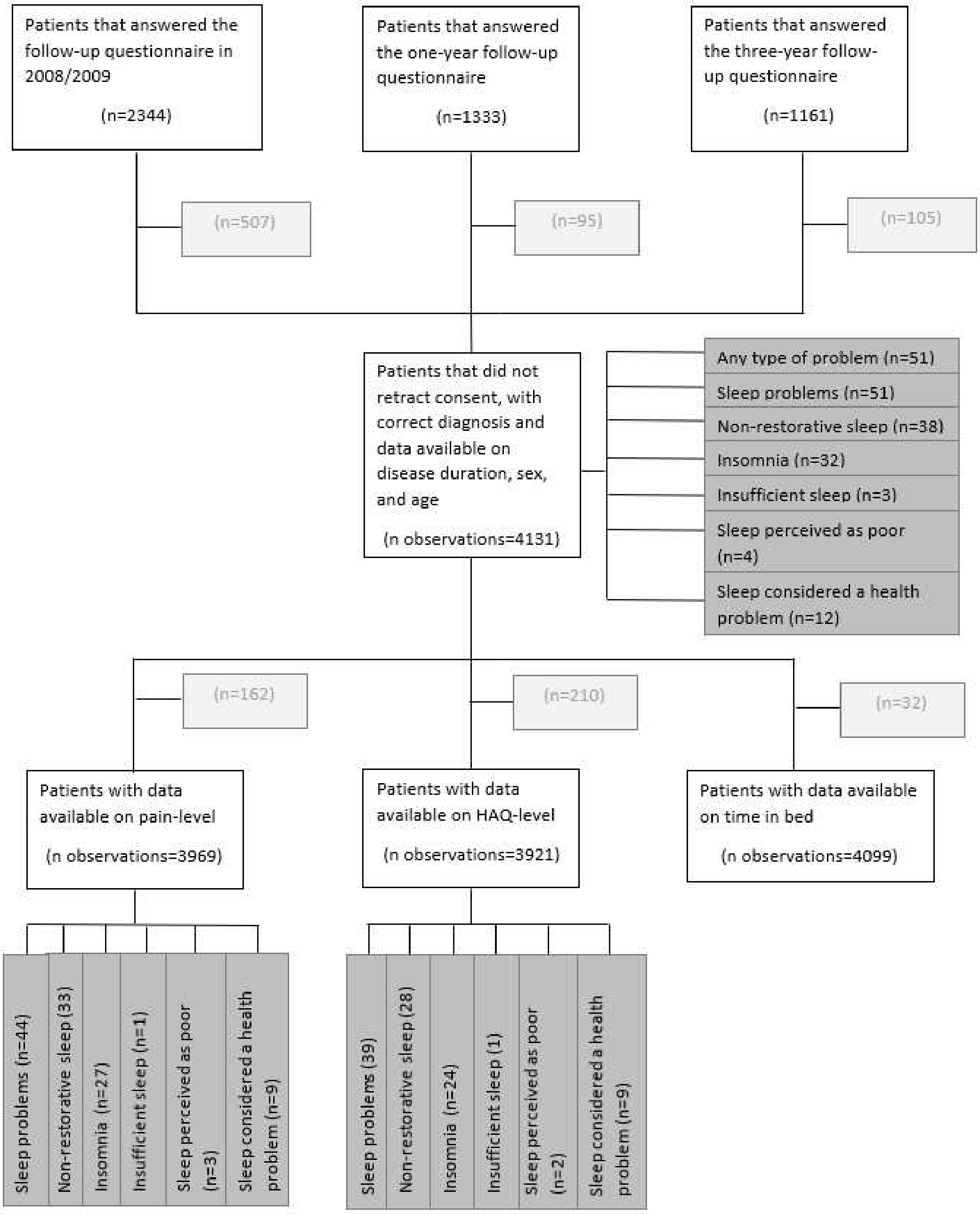

Figure S2: Time in bed (proportion of short, normal, long) for full cohort stratified by disease duration for 4131 observations from 3265 individuals newly diagnosed with RA during 1996-2018 in Sweden.

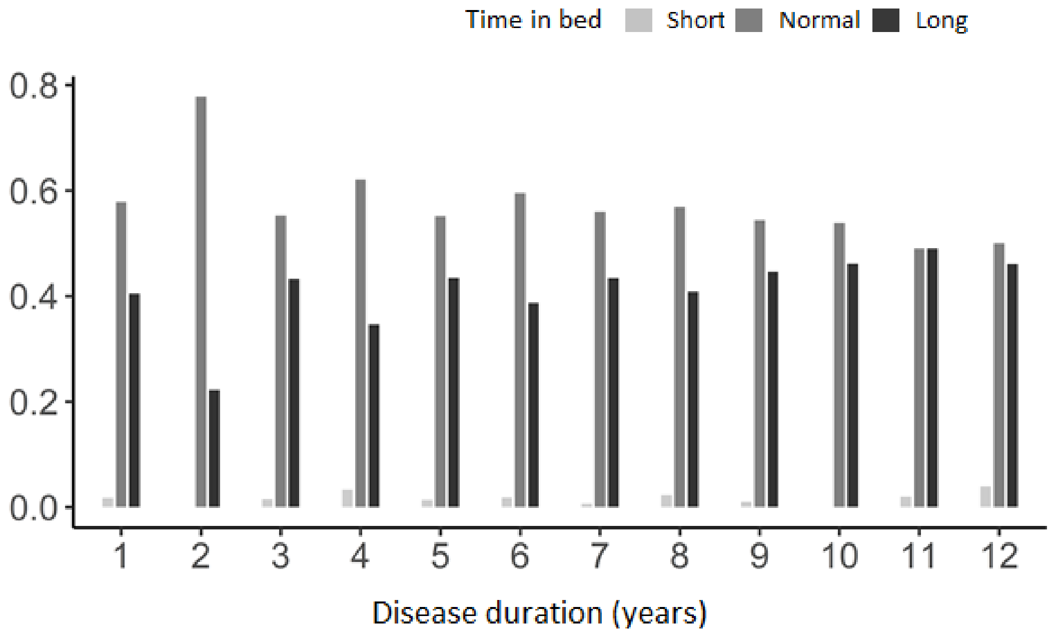

Figure S3: Proportion experiencing problems with each sleep domain stratified by 10-year interval age groups for 4131 observations from 3265 individuals newly diagnosed with RA during 1996-2018 in Sweden.

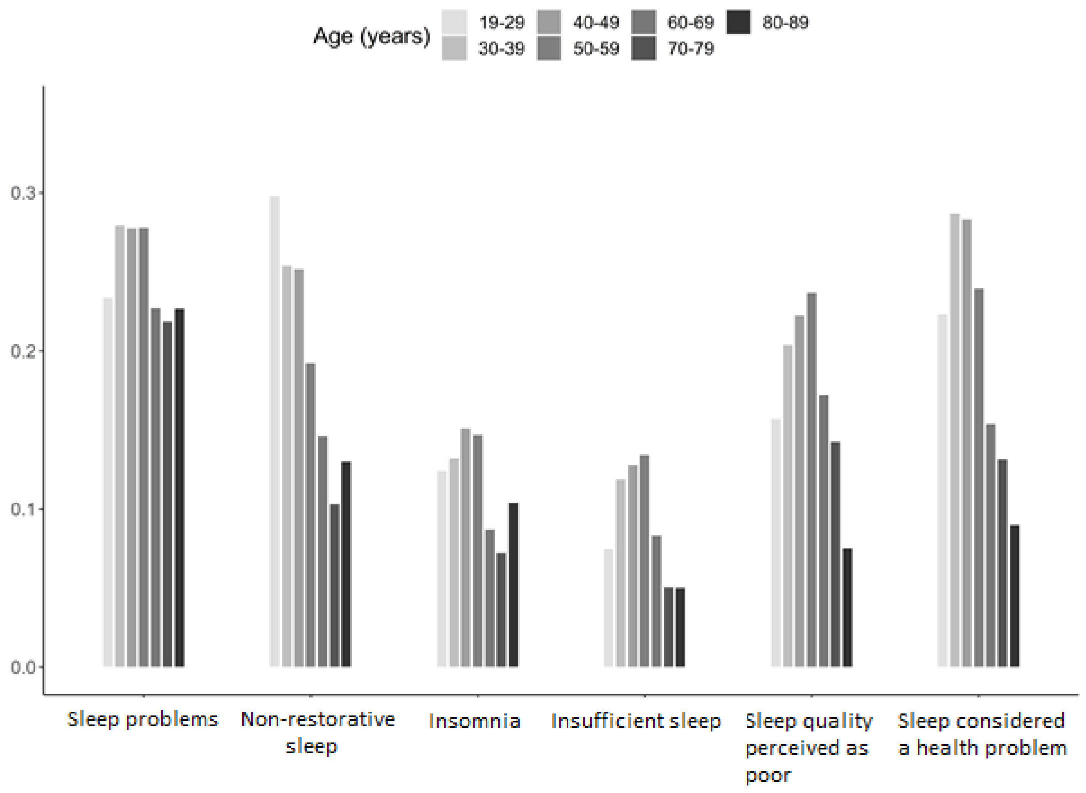

Figure S4: Proportion of patients experiencing problems with each sleep domain stratified sex for 4131 observations from 3265 individuals newly diagnosed with RA during 1996-2018 in Sweden.

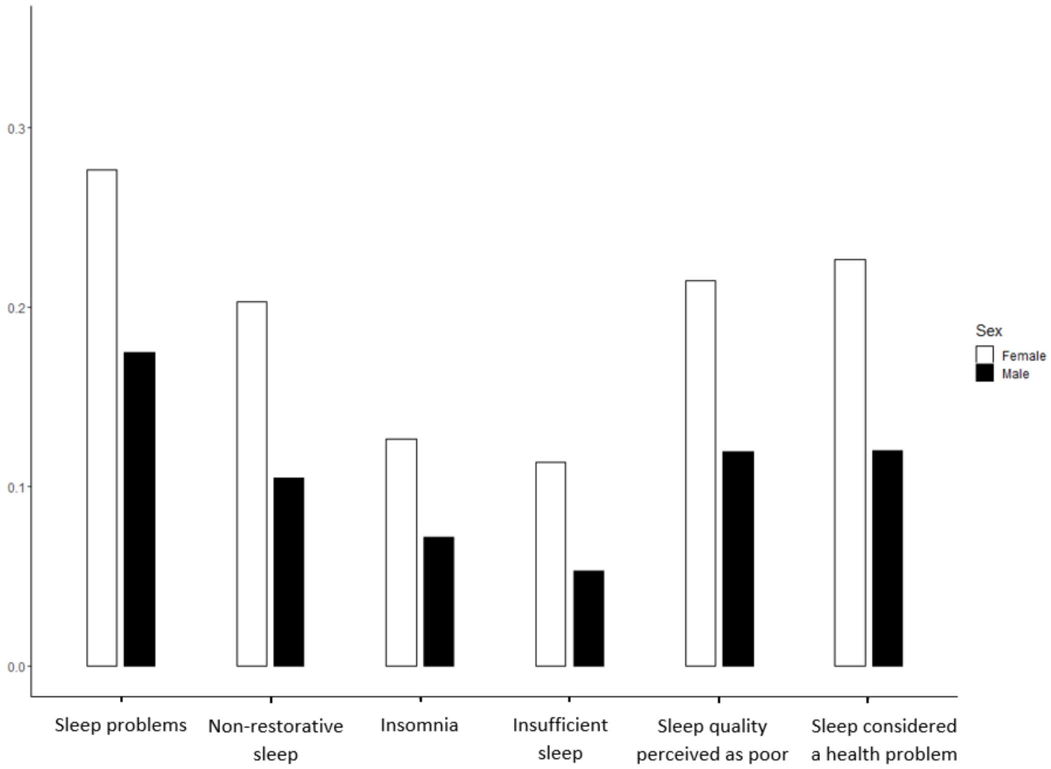

Supplement: Supplementary data [file rmdopen-2021-001800supp001.pdf]
